# A Mendelian randomization study of the effect of calcium on coronary artery disease, myocardial infarction and their risk factors

**DOI:** 10.1038/srep42691

**Published:** 2017-02-14

**Authors:** Lin Xu, Shi Lin Lin, C. Mary Schooling

**Affiliations:** 1 School of Public Health, Li Ka Shing Faculty of Medicine, The University of Hong Kong, Hong Kong SAR, China; 2 School of Public Health, Sun Yat-sen University, Guangzhou, Guangdong Province, China; 3 School of Urban Public Health, Hunter College and CUNY School of Public Health, New York, New York, USA

## Abstract

Meta-analyses of randomized controlled trials (RCTs) suggest calcium could have adverse effects on cardiovascular disease, although these findings are controversial. To clarify, we assessed whether people with genetically higher calcium had a higher risk of coronary artery disease (CAD), myocardial infarction (MI) and their risk factors. We used a two-sample Mendelian randomization study. We identified genetic variants (single nucleotide polymorphisms (SNPs)) that independently contributed to serum calcium at genome-wide significance which we applied to large extensively genotyped studies of CAD, MI, diabetes, lipids, glycaemic traits and adiposity to obtain unconfounded estimates, with body mass index (BMI) as a control outcome. Based on 4 SNPs each 1 mg/dl increase in calcium was positively associated with CAD (odds ratio (OR) 1.49, 95% confidence interval (CI) 1.02–2.17), MI (OR 1.58, 95% CI 1.06–2.35), LDL-cholesterol (0.21 standard deviations, 95% CI 0.01–0.4), total cholesterol (0.21 standard deviations, 95% CI 0.03-0.38) and possibly triglycerides (0.19 standard deviations, 95% CI −0.1–0.48), but was unlikely related to BMI although the estimate lacked precision. Sensitivity analysis using 13 SNPs showed a higher risk for CAD (OR 1.87, 95% CI 1.14–3.08). Our findings, largely consistent with the experimental evidence, suggest higher serum calcium may increase the risk of CAD.

Calcium is widely seen as part of a healthy diet that promotes bone health. Recent meta-analysis of randomized controlled trials (RCTs) has suggested that calcium has little benefit for fracture prevention^1^. Unexpectedly, some meta-analyses of these RCTs also suggest that calcium may increase the risk of cardiovascular events, myocardial infarction (MI) and stroke^2^,^3^,^4^,^5^, although not all meta-analyses of RCTs concur on this point^6^. Controversy over calcium supplements arose with the Auckland Calcium Study (ACS) reporting calcium supplements increased cardiovascular disease (CVD) events in a secondary analysis of a large RCT in 2008^7^. However, this was a hypothesis generating study because CVD events were not one of the original study outcomes. Re-analyzing data from the US Women’s Health Initiative (WHI), in a sub-group analysis of those not taking calcium supplements at randomization, the same authors of the ACS found that calcium supplementation increased the risk of MI^2^. Incorporating the results in a meta-analysis, the use of calcium supplementation increased the risk of MI, by 21% 2. This meta-analysis only included a small number of trials (n = 7) and compliance was low especially in the calcium arm due to adverse effects on gastrointestinal disorders, which might attenuate the potential impact on CVD events. Moreover, none of these trials were specifically designed to include primary CVD endpoints, and thus CVD events were not collected in a standard and systematic manner. In 2015, another meta-analysis of RCTs found the risk of coronary heart disease (CHD) in older women was not increased by calcium supplements^6^. An increase in CVD events in earlier studies was attributed to selection bias^6^, however, all these studies relied on adverse event reporting, whose classification is open to biases, because CVD events may not be considered as intervention-related and were not reported in some trials^8^. Another two recent meta-analysis of RCTs demonstrated calcium supplements (with or without vitamin D) increased risk of MI by 24–28% 4,9. The discrepancy beteen meta-analyses may be due to the inclusion of controversial RCTs, such as trials with participants who differed on CVD risk between study arms at baseline^10^. Moreover, meta-analysis of RCTs also suggest inconsistent effects on cardiovascular disease risk factors, specifically that calcium reduces the risk of type 2 diabetes (T2DM)^2^,^3^,^4^,^5^ and improves glucose metabolism but also increases lipids^11^,^12^. These contradictory findings for different cardiovascular risk factors again make interpretation uncertain. A large RCT with cardiovascular events as the primary endpoint and its risk factors as secondary outcomes would be definitive, but would take several years and might be difficult to justify given the lack of convincing evidence for benefits of calcium supplementation, even for bone mineral density^1^.

In this situation assessing coronary artery disease (CAD), MI and their risk factors according to genetically determined serum calcium, i.e., Mendelian randomization (MR), may provide insight. Since genetic endowment is randomly allocated at conception MR studies provide genetic randomization analogous to the randomization in RCTs, and so are less vulnerable to confounding and reverse causation. MR has been successfully used in cardiovascular research to investigate potential etiological mechanisms, prioritize drug targets and increase understanding of current therapies^13^. Here, we took advantage of genome wide association studies of calcium and large extensively genotyped studies of CAD, MI, T2DM and other CVD risk factors to obtain less confounded estimates of the effect of serum calcium on CAD, MI and their risk factors. Given calcium has no effect on body mass index (BMI) in meta-analysis of RCTs^14^,^15^, we used BMI as a control outcome, because in an unbiased analysis we would expect to see no association of calcium with BMI.

## Methods

### Data sources

#### Serum calcium

From a large meta-analysis of genome wide associations studies (GWAS) including 20,611 individuals of European ancestry^16^, we obtained single nucleotide polymorphisms (SNPs) independently contributing to serum calcium at genome wide significance (p < 5 × 10^−8^). We assessed correlation (linkage disequilibrium) between SNPs using SNP Annotation and Proxy (SNAP) Search system (http://www.broadinstitute.org/mpg/snap/ldsearchpw.php) for the same reference catalog and population^17^. When the correlation coefficient between SNPs was high (R^2^ ≥ 0.8) we discarded the SNP with the larger P value, when the correlation was lower we kept all SNPs but took into account their correlation matrix. We identified pleiotropic effects of these SNPs from Ensembl (Homo sapiens – phenotype) (http://grch37.ensembl.org/Homo_sapiens/Info/Index), a comprehensive genotype to phenotype cross-reference. We used SNPs that are approximately independent as determinants of calcium (i.e., R^2^ < 0.01) as the main analysis. As a sensitivity analysis we further included SNPs with low correlation (0.01 ≤ R^2^ < 0.8), identified from the linkage disequilibrium correlation matrix (Supplementary Table 1).

#### Coronary artery disease and its risk factors

Association of SNPs with the phenotypes were extracted from publicly available consortia. Data on coronary artery disease/myocardial infarction have been contributed by Coronary ARtery DIsease Genome wide Replication and Meta-analysis (CARDIoGRAM) plusC4D investigators and have been downloaded from www.CARDIOGRAMPLUSC4D.ORG^18^. As no calcium-related SNPs can be identified from the CARDIoGRAMplusC4D Metabochip, all summary data on the gene-CAD association were obtained from the CARDIoGRAM GWAS, a meta-analysis of 22 GWAS studies of European descent imputed to HapMap 2 involving 22,233 cases and 64,762 controls. As sensitivity analysis, we also use CARDIoGRAMplusC4D 1000 Genomes-based GWAS, a meta-analysis of GWAS studies of mainly European, South Asian, and East Asian, descent imputed using the 1000 Genomes phase 1 v3 training set with 38 million variants^19^. The study interrogated 9.4 million variants and involved 60,801 coronary artery disease (CAD) cases and 123,504 controls, and 43,676 myocardial infarction (MI) cases and 128,199 controls^19^. Data on T2DM was contributed by the DIAbetes Genetics Replication And Meta-analysis (DIAGRAM, http://diagram-consortium.org/downloads.html), which includes 12,171 cases and 56,862 controls in Stage 1 GWAS^20^ and 26,488 cases and 83,964 controls in the Trans-ethnic GWAS meta-analysis^21^,^22^. Genetic associations with high density lipoprotein (HDL) cholesterol, low density lipoprotein (LDL) cholesterol, triglycerides, and total cholesterol in 188,577 people have been contributed by Global Lipids Genetics Consortium (GLGC) investigators and have been downloaded from http://csg.sph.umich.edu/abecasis/public/lipids2013/22. Genetic associations with fasting insulin (n = 38,238), fasting glucose (n = 46,186), log-transformed homeostatic model assessment insulin resistance (log-transformed HOMA-IR, n = 46,186) have been contributed by Meta-Analyses of Glucose and Insulin-related traits Consortium (MAGIC) investigators and have been downloaded from http://www.magicinvestigators.org/, which relates to people of European ancestry without diabetes^23^.

#### Control outcome

We used BMI as a control outcome that, based on RCTs, should be unrelated to serum calcium^14^,^15^. Genetic associations with BMI (kg/m^2^) have been contributed by The Genetic Investigation of ANthropometric Traits (GIANT) investigators and have been downloaded from https://www.broadinstitute.org/collaboration/giant/index.php/GIANT_consortium_data_files which has BMI for 152,893 men and 171,977 women of European ancestry^24^.

#### Statistical analysis

SNP-specific Wald estimates (ratio of SNP on outcome to SNP on calcium) of the effect of calcium on each outcome were combined using weighted generalized linear regression to account for correlation between the SNPs^25^, giving an odds ratio (OR) for CAD, MI and T2DM, and regression coefficients (β) for the other outcomes with 95% confidence interval (CI). As a sensitivity analysis we used inverse variance weighted (IVW) estimator with fixed effects^26^. Provided that the genetic variants are uncorrelated, the IVW estimate is asymptotically equal to the two-stage least squares estimate commonly used with individual-level data^27^. In IVW, the ratio estimates from each IV (or SNP) are combined in an inverse-variance weighted estimator. We also used a weighted median method to combine the SNP specific estimates for the uncorrelated SNPs^25^. Even after excluding SNPs with known potentially pleiotropic effects (i.e., SNPs that could influence both serum calcium and CAD/CVD risk factors), the estimates could still be biased by unknown pathways that directly link the genetic determinants of calcium to CAD, MI or other risk factors independent of the pathway via calcium. To assess this possible bias, MR-Egger regression was also used^28^. The same as the IVW method, MR Egger also uses an inverse-variance weighted estimator. It differs from the IVW method in that it allows the intercept (pleiotropy effect) to be non-zero. If the intercept in the regression model in MR-Egger were truly zero (or were constrained to be zero), the MR-Egger slope estimate is the same as the regression coefficient from IVW. If the intercept is zero it suggests that there is no violation of the exclusion restriction criteria (i.e., no horizontal pleiotropy); it provides an estimate of the average pleiotropic effect across all of the genetic variants, because it reflects the effect of the joint instruments on outcome (e.g., CAD/MI) when there is zero effect of the genetic variants on the risk factor (e.g. calcium). An intercept term that differs from zero suggests horizontal pleiotropy and that the IVW estimate may be biased. The weakness of the instruments was evaluated using the first-stage F-statistics calculated by 
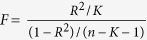
, where R^2^ indicates the variance explained by each genetic instrument, K indicated the number of instrument, and n indicate the sample size of the first stage^29^. All statistical analysis was performed using STATA 13.1 and R-software (Version 3.2.5).

## Results

### Genetic determinants of serum calcium

GWAS gave 128 SNPs related to serum calcium with p < 1 × 10^−5^ 16,30, from which we excluded 84 SNPs that do not reach genome wide significance (p < 5 × 10^−8^), 29 highly correlated SNPs (R^2^ ≥ 0.8), and 2 SNPs with pleiotropic effects (on bilirubin and parathyroid hormone) giving 13 genome-wide significant SNPs (rs4306808 in *FAM162A*, rs7336933 near *DGKH/KIAA0564*, rs17711722 in *VKORC1L1*, rs1067 in *WDR5B*; rs17267388 and rs11929034 in *PARP9*, rs4491840 in *CCDC58*, rs9864290 in *CSTA*, rs10491003 (closest gene *GATA3*), and rs16832956, rs17251221, rs13095172 and rs10222633 in *CASR*) as shown in Table 1. For the 13 selected SNPs, 9 were correlated (R^2^ > 0.1) among each other according to SNAP with HapMap release 22, as shown in the correlation matrix in Supplementary Table 1. Of these 13 SNPs, 4 from 4 different genes (rs7336933 (*DGKH/KIAA0564),* rs17711722 (*VKORC1L1),* rs17251221 (*CASR)* and rs10491003 (*GATA3*)) were uncorrelated R^2^ < 0.05 in HapMap CEU population. The first-stage F-statistics for the IV including these 4 SNPs was 52.

### Association of genetically determined serum calcium concentrations with CAD risk

Table 2 shows that the estimates for the causal effect of 1 mg/dl higher serum calcium were consistently in the direction of higher risk of CAD and MI based on 4 SNPs, although sometimes the lower limit of the confidence interval included the null value in both CARDIoGRAM (OR 1.62, 95% CI 0.86 to 3.07) and CARDIoGRAMplueC4D 1000 Genomes-based GWAS (OR 1.49, 95% CI 1.02 to 2.17 for CAD, and 1.58, 95% CI 1.06 to 2.35 for MI) using IVW or using a weighted median (1.66, 95% CI 1.12 to 1.81 for CAD, and 1.65, 95% CI 1.06 to 2.56 for MI). The results were consistent based on 13 SNPs in CARDIoGRAM (OR 1.87, 95% CI 1.14 to 3.08) and CARDIoGRAMplueC4D 1000 Genomes-based GWAS using IVW (1.25, 95% CI 0.92 to 1.70 for CAD, and 1.32, 95% CI 0.94 to 1.85 for MI).

### Association of genetically determined calcium with CAD risk factors

In all analyses the estimated effect of calcium on BMI was null but lacked precision. Similar observations from results using 4 SNPs were found for T2DM (OR 1.34, 95% CI 0.65 to 2.74) and glycemic parameters including fasting glucose, insulin and log HOMA-IR. In contrast, based on 4 SNPs, the estimates for the causal effect of 1 mg/dl higher serum calcium on LDL-cholesterol (0.21 standard deviation, 95% CI 0.01 to 0.40) and total cholesterol (0.29 standard deviation, 95% CI 0.09 to 0.48) were positive but in sensitivity analysis the confidence interval included the null for triglycerides (0.19 standard deviation, 95% CI −0.1 to 0.48). Using the MR-Egger method with the 4 SNPs, we could not reject the hypothesis that an association of these 4 SNPs with CAD, MI, T2DM or their risk factors was not independent of the effects on calcium (Supplementary Table 2).

## Discussion

Consistent with most previous meta-analyses of RCTs^2^,^3^,^4^,^5^, we found that higher serum calcium was associated with a higher risk of CAD and probably MI, although we cannot definitively rule out the possibility of no effect. Findings for CAD risk factors, LDL- and total cholesterol were also positive, consistent with previous RCTs^11^,^12^. In addition, as expected, calcium appeared not to be associated with BMI^14^,^15^. As such, our study replicates findings from RCTs and extends them by showing the same pattern of associations for endogenous calcium in very large studies including substantial numbers of men as well as of women.

Our findings for CAD are consistent with most previous meta-analyses of RCTs largely pertaining to women^2^,^3^,^4^,^6^,^9^, although the estimate is higher than that suggested by these RCTs, which could be because calcium has a stronger association with MI in men. However, MR is more suitable for establishing direction than exact effect sizes, because genetically determined calcium represents lifetime exposure, whereas the RCTs were relatively short in duration and potentially biased towards the null by non-compliance, if the participants were successfully blinded. More generally, our findings are consistent with the observation that countries with higher calcium intake, such as Northern European countries, also have higher CAD mortality rates, while countries with low calcium intake tend to have low CAD mortality, such as China, Korea and Japan^31^. Of course, ecological evidence never proves causality, but this observation does require some explanation.

A previous Mendelian randomization study, using 17 SNPs for calcium from in and around the CASR gene region obtained from ~7000 participants in the European Prospective Investigation into Cancer and Nutrition (EPIC) -InterAct study, found calcium associated with higher fasting glucose in MAGIC^32^. However, our estimate, with genetically determined calcium obtained from a GWAS with a larger sample (n = 20,611)^16^, gave no association of calcium with fasting glucose (beta = −0.001, 95% CI −0.14 to 0.14). We also cannot completely rule out the possibility that the result on glucose was due to an insufficient sample size. A post-hoc power calculation for specifically glucose assuming a statistical confidence level of 0.05, an effect size of 0.03 (as results from the analysis using 13 SNPs), and serum calcium and glucose levels from the GWAS^16^,^23^ showed power of less than 20%, suggesting larger MR studies are necessary to further clarify the effect on fasting glucose. Our results are consistent with previous RCTs showing calcium supplementation has no effects on fasting glucose, although calcium supplements intake tends to reduce fasting insulin and improve insulin resistance^11^,^12^.

The heritability for total calcium is between 33% and 78% in twin studies^33^,^34^, suggesting that serum calcium levels are tightly regulated. Three major hormones are involved in the regulation of calcium homeostasis, parathyroid hormone (PTH), calcitonin and 1,25-dihydroxyvitamin D, which act on their corresponding receptors in bone, gut and kidney to maintain serum calcium concentrations^35^. A key regulator of the PTH release is the calcium-sensing receptor (CASR), which is mainly in the plasma membrane of chief cells of the parathyroid gland and in cells of the renal tubule^36^. The CASR gene encodes a protein which binds to calcium and thus plays an essential role in calcium homeostasis^37^. Apart from CASR, another gene, GATA3 encodes a GATA transcription factor involved in T cell lymphopoiesis, renal and vestibular morphogenesis, and parathyroid gland development^38^. Functional studies have shown that GATA3 haploinsufficiency causes hypoparathyroidism in populations of different ethnicities^39^,^40^,^41^,^42^. Other mechanisms exist by which calcium might contribute to CAD, for example by promoting carotid intima-media thickness^43^, coronary artery calcification, as occurs with calcium based phosphate binders versus non-calcium based phosphate binders^44^, and coagulation^45^. Acute induction of severe hypercalcemia in animal models reduces blood clotting time by 50%^46^. *In vitro*, increasing calcium concentrations across the physiological range reduces the clotting time of human blood^47^. These mechanisms could underlie effects of calcium on CAD.

Several methodologic considerations and limitations bear discussion. First, the genetic variants used for genetically determined calcium were all strongly related to calcium at GWAS level significance. No obvious reason exists for the existence of confounders of the association of these genetic variants with the outcomes considered here, for example by population stratification, because the underlying studies relate to relatively ethnically homogeneous populations of mainly European ancestry. Both calcium levels and CVD rates vary across Europe, which could be due to other factors determining calcium and CVD^31^, of which the calcium related genetic variants are only a marker. However, the estimate was dominated by genetic variants from the calcium-sensing receptor (CASR) gene functionally relevant to calcium, making such confounding unlikely. Second, the genetic variants used are not known to be associated with other phenotypes that might influence CAD and its risk factors, thus making biases from direct associations of SNPs with the outcomes, i.e., “pleiotropy” or violation of the “exclusion-restriction” assumption, unlikely. Moreover, we found no evidence of directional pleiotropy, i.e. that the genetic variants used to predict calcium had effects on CAD or its risk factors independent of effects via calcium. Third, we replicated established experimental evidence from meta-analysis of RCTs^14^,^15^ by showing that calcium had no effect on BMI, which gives more credence to the other estimates using the same genetic determinants of calcium. Fourth, given the use of summarized data in two samples, serum calcium levels were not measured in the sample with the outcome. However, two-sample instrumental variable analysis is more robust to chance associations than analysis of a single sample^48^. Fifth, it is not be possible to perform sub-group analysis or multivariable analysis as rigorously in two-sample MR as in one-sample MR using individual-level data. For example, whether the SNPs for serum calcium have different effects on CAD, T2DM or other CVD risk factors at different levels of serum calcium, by sex or at different ages could not be tested, and whether there was significant heterogeneity among sub-populations for individual instruments could not be assessed. In addition, there might be participant overlap in this two-sample MR (i.e., the same data used both for SNP selection and to calculate the IV effect)^49^. However, given the very large sample size of the CARDIoGRAMplusC4D consortium, assuming a 50% overlapping the sample overlap in this study is only 12%, because the dataset for deriving calcium related SNP was much smaller (n = 20,611). Thus bias from sample overlapping, if any, should not be a major concern^50^. Finally, both IVW and MR-Egger methods use weights that under the “NO Measurement Error (NOME) assumption”^51^, that is assuming the SNP-exposure associations to be known, rather than estimated. This assumption cannot be tested directly^51^. However, we used I^2^ statistics to quantify the strength of NOME violation for MR-Egger and did not find significant evidence of the violation.

Our study indicates that genetically higher serum calcium concentrations could have a harmful effect on MI and CAD. On the precautionary principle, given calcium does not seem as important in bone health as thought, our findings suggest reconsideration of the use of calcium supplementation and particularly fortification in the general population, especially in products used by older people who have higher risk of CAD.

## Additional Information

**How to cite this article:** Xu, L. *et al*. A Mendelian randomization study of the effect of calcium on coronary artery disease, myocardial infarction and their risk factors. *Sci. Rep.* **7**, 42691; doi: 10.1038/srep42691 (2017).

**Publisher's note:** Springer Nature remains neutral with regard to jurisdictional claims in published maps and institutional affiliations.

## Supplementary Material

Supplementary Table 1 and 2

## Figures and Tables

**Table 1:** Characteristics of the single nucleotide polymorphisms (SNP) used for genetically determined serum calcium.

SNP	Nearest gene	Effect allele	Other allele	Effect^†^	Standard error	MAF	R^2^	Main analysis
rs4306808	FAM162A	G	C	0.054	0.0084	0.1675	0.08	
rs7336933	DGKH/KIAA0564	G	A	0.022	0.0040	0.1248	0.01	√
rs17711722	VKORC1L1	T	C	0.021	0.0030	0.4355	0.02	√
rs17267388	PARP9	A	G	0.036	0.0059	0.1300	0.03	
rs1067	WDR5B	A	G	0.033	0.0059	0.1444	0.03	
rs13095172	CSTA;CASR	T	C	0.028	0.0046	0.3524	0.04	
rs11929034	PARP9	A	G	0.038	0.0063	0.1312	0.03	
rs4491840	CCDC58	A	G	0.042	0.0060	0.1673	0.05	
rs16832956	CSTA;CASR	G	C	0.044	0.0053	0.1641	0.05	
rs17251221	CASR	G	A	0.061	0.0063	0.0942	0.06	√
rs10222633	CASR	G	A	0.030	0.0042	0.3852	0.04	
rs10491003	GATA3	T	C	0.027	0.0050	0.1040	0.01	√
rs9864290	CSTA	C	T	0.028	0.0047	0.4453	0.04	

MAF: minor allele frequency.

^†^Increase in calcium (mg/dl) per effect allele.

**Table 2:** Mendelian randomization estimates of the causal association of serum calcium (mg/dl) with coronary artery disease (CAD), myocardial infarction (MI) and their risk factors.

	Main analysis (4 SNPs)	Weighted-median	Sensitivity analysis (13 SNPs)
	IVW		WGL regression
	OR (95% CI)	OR (95% CI)	OR (95% CI)
CAD (CARDIoGRAM)			
CARDIoGRAM GWAS	1.62 (0.86 to 3.07)	1.48 (0.80 to 2.71)	1.87 (1.14 to 3.08)^**^
1000 Genomes	1.49 (1.02 to 2.17)^*^	1.66 (1.12 to 1.81)^**^	1.25 (0.92 to 1.70)
1000 Genomes –MI	1.58 (1.06 to 2.35)^*^	1.65 (1.06 to 2.56)^*^	1.32 (0.94 to 1.85)
T2DM			
DIAGRAM GWAS	1.34 (0.65 to 2.74)	1.31 (0.62 to 2.8)	1.64 (0.87 to 3.10)
Trans-ethnic GWAS meta-analysis	1.21 (0.71 to 2.07)	1.31 (0.71 to 2.5)	1.06 (0.65 to 1.75)
	β (95% CI)	β (95% CI)	β (95% CI)
BMI, SD (1 SD = 4.77 kg/m^2^)	0.07 (−0.1 to 0.25)	0.04 (−0.12 to 0.2)	0.09 (−0.04 to 0.22)
LDL-C, SD (1 SD = 38.7 mg/dL)	0.21 (0.01 to 0.40)^*^	0.20 (−0.03 to 0.43)	0.15 (−0.02 to 0.33)
HDL-C, SD (1 SD = 15.5 mg/dL)	0.07 (−0.11 to 0.25)	0.09 (−0.11 to 0.3)	0.04 (−0.12 to 0.21)
TG, SD (1 SD = 90.7 mg/dL)	0.19 (−0.1 to 0.48)	0.12 (−0.09 to 0.33)	0.19 (0.03 to 0.35)^*^
TC, SD (1 SD = 41.8 mg/dL)	0.29 (0.09 to 0.48)^*^	0.30 (0.08 to 0.51)^*^	0.21 (0.03 to 0.38)^*^
Fasting glucose, mmol/l	−0.001(−0.14 to 0.14)	−0.01(−0.16 to 0.14)	0.03 (−0.09 to 0.15)
Fasting insulin, log pmol/l	−0.10 (−0.33 to 0.13)	−0.04 (−0.21 to 0.13)	−0.09 (−0.21 to 0.04)
Log HOMA-IR	−0.09 (−0.31 to 0.13)	−0.03 (−0.2 to 0.14)	−0.06 (−0.20 to 0.07)

WGL: weighted generalized linear; IVW: inverse variance weighted; SD: standard deviation; OR: odds ratio; CI: confidence interval; T2DM: type-2 diabetes mellitus; BMI: body mass index; LDL-C: low-density lipoprotein cholesterol; HDL-C: high-density lipoprotein cholesterol; TG: triglycerides; TC: total cholesterol; HOMA-IR: homeostatic model assessment of insulin resistance ^*^P < 0.05; ^**^P < 0.01.
