# Supplementary material for: A Mendelian randomization study of the effect of calcium on coronary artery disease, myocardial infarction and their risk factors
Source: Sci Rep. 2017 Feb 14;7:42691. doi: 10.1038/srep42691 (PMC5307362; doi:10.1038/srep42691)
Supplement: Supplementary Table 1 and 2 [file srep42691-s1.doc]

**A Mendelian randomization study of the effect of calcium on coronary artery disease, myocardial infarction and their risk factors**

Lin Xu, PhD^1*^, Shi Lin Lin, PhD ^1^, C Mary Schooling, PhD ^1,2^

^1^ School of Public Health, Li Ka Shing Faculty of Medicine, The University of Hong Kong, Hong Kong SAR, China

^2^ School of Urban Public Health, Hunter College and CUNY School of Public Health, New York, New York, USA

Short title: Mendelian randomization of calcium and coronary artery disease

Corresponding author: Dr. L Xu

Patrick Manson Building

7 Sassoon Road, Hong Kong

Tel: (852) 3917 6732

Fax: (852) 2855 9528

Email: [linxu@hku.hk](mailto:linxu@hku.hk)

Supplementary Table 1. Correlation matrix for the selected calcium-related SNPs.

|  | rs10222633 | rs10491003 | rs1067 | rs11929034 | rs13095172 | rs16832956 | rs17251221 | rs17267388 | rs17711722 | rs4306808 | rs4491840 | rs7336933 | rs9864290 |
| --- | --- | --- | --- | --- | --- | --- | --- | --- | --- | --- | --- | --- | --- |
| rs10222633 | 1.00 | 0.00 | 0.25 | 0.31 | 0.67 | 0.44 | 0.41 | 0.38 | 0.00 | 0.29 | 0.31 | 0.00 | 0.61 |
| rs10491003 | 0.00 | 1.00 | 0.00 | 0.00 | 0.00 | 0.00 | 0.00 | 0.00 | 0.00 | 0.00 | 0.00 | 0.00 | 0.00 |
| rs1067 | 0.25 | 0.00 | 1.00 | 0.60 | 0.38 | 0.42 | 0.54 | 0.70 | 0.00 | 0.52 | 0.60 | 0.00 | 0.34 |
| rs11929034 | 0.31 | 0.00 | 0.60 | 1.00 | 0.39 | 0.71 | 0.69 | 0.89 | 0.00 | 0.49 | 0.77 | 0.00 | 0.55 |
| rs13095172 | 0.67 | 0.00 | 0.38 | 0.39 | 1.00 | 0.48 | 0.61 | 0.36 | 0.00 | 0.32 | 0.50 | 0.00 | 0.74 |
| rs16832956 | 0.44 | 0.00 | 0.42 | 0.71 | 0.48 | 1.00 | 0.84 | 0.68 | 0.00 | 0.48 | 0.71 | 0.00 | 0.74 |
| rs17251221 | 0.41 | 0.00 | 0.54 | 0.69 | 0.61 | 0.84 | 1.00 | 0.67 | 0.00 | 0.58 | 0.85 | 0.00 | 0.67 |
| rs17267388 | 0.38 | 0.00 | 0.70 | 0.89 | 0.36 | 0.68 | 0.67 | 1.00 | 0.00 | 0.64 | 0.74 | 0.00 | 0.52 |
| rs17711722 | 0.00 | 0.00 | 0.00 | 0.00 | 0.00 | 0.00 | 0.00 | 0.00 | 1.00 | 0.00 | 0.00 | 0.00 | 0.00 |
| rs4306808 | 0.29 | 0.00 | 0.52 | 0.49 | 0.32 | 0.48 | 0.58 | 0.64 | 0.00 | 1.00 | 0.67 | 0.00 | 0.36 |
| rs4491840 | 0.31 | 0.00 | 0.60 | 0.77 | 0.50 | 0.71 | 0.85 | 0.74 | 0.00 | 0.67 | 1.00 | 0.00 | 0.55 |
| rs7336933 | 0.00 | 0.00 | 0.00 | 0.00 | 0.00 | 0.00 | 0.00 | 0.00 | 0.00 | 0.00 | 0.00 | 1.00 | 0.00 |
| rs9864290 | 0.61 | 0.00 | 0.34 | 0.55 | 0.74 | 0.74 | 0.67 | 0.52 | 0.00 | 0.36 | 0.55 | 0.00 | 1.00 |

Supplementary Table 2. Log odds ratio of coronary heart disease, myocardial infarction, type 2 diabetes mellitus, and their risk factors per calcium-raising allele.

|  | **I^2^ statistics, %** | **P values from Q-test** | **Egger_intercept^†^** | **Standard error** | **p-value** |
| --- | --- | --- | --- | --- | --- |
| **CHD** |  |  |  |  |  |
| CARDIoGRAM | 25 | 0.26 | -0.013 | 0.028 | 0.683 |
| CARDIoGRAMplusC4D 1000 Genomes-based GWAS | 8 | 0.36 | -0.018 | 0.015 | 0.347 |
| CARDIoGRAMplusC4D –myocardial infarction | 0 | 0.63 | -0.006 | 0.017 | 0.76 |
| **T2DM** |  |  |  |  |  |
| DIAGRAM GWAS | 0 | 0.89 | -0.019 | 0.028 | 0.561 |
| Trans-ethnic GWAS | 0 | 0.78 | -0.018 | 0.021 | 0.479 |
| BMI, SD (1 SD=4.77 kg/m2) | 37 | 0.19 | 0.011 | 0.005 | 0.174 |
| LDL-C, SD (1 SD= 38.7 mg/dL) | 0 | 0.64 | -0.003 | 0.008 | 0.749 |
| HDL-C, SD (1 SD= 15.5 mg/dL) | 0 | 0.81 | -0.004 | 0.007 | 0.636 |
| Triglycerides, SD (1 SD= 90.7 mg/dL) | 61 | 0.05 | 0.011 | 0.011 | 0.400 |
| Total cholesterol, SD (1 SD= 41.8 mg/dL) | 0 | 0.94 | -0.001 | 0.007 | 0.865 |
| Fasting glucose, mmol/l | 0 | 0.99 | 0.0007 | 0.005 | 0.901 |
| Fasting insulin, log pmol/l | 64 | 0.04 | -0.010 | 0.008 | 0.374 |
| Log HOMA-IR | 52 | 0.10 | -0.009 | 0.008 | 0.403 |

**^†^**Intercept term represents the average association per calcium-raising allele with the outcomes in the absence of association with the serum calcium.
